# Variations in Umbilical Cord Hematopoietic and Mesenchymal Stem Cells With Bronchopulmonary Dysplasia

**DOI:** 10.3389/fped.2019.00475

**Published:** 2019-11-14

**Authors:** Sonali Chaudhury, Juanita Saqibuddin, Robert Birkett, Kate Falcon-Girard, Morey Kraus, Linda M. Ernst, William Grobman, Karen K. Mestan

**Affiliations:** ^1^Division of Hematology/Stem Cell Transplant, Department of Pediatrics, Ann & Robert H. Lurie Children's Hospital of Chicago, Northwestern University Feinberg School of Medicine, Chicago, IL, United States; ^2^Division of Neonatology, Department of Pediatrics, Ann & Robert H. Lurie Children's Hospital of Chicago, Northwestern University Feinberg School of Medicine, Chicago, IL, United States; ^3^ViaCord LLC, A Perkin Elmer Company, Cambridge, MA, United States; ^4^Department of Pathology, NorthShore University, Evanston, IL, United States; ^5^Department of Obstetrics & Gynecology and Maternal Fetal Medicine, Northwestern University Feinberg School of Medicine, Chicago, IL, United States

**Keywords:** premature infant, neonatal outcome, cord blood, stem cells, cytokines, bronchopulmonary dysplasia

## Abstract

**Objective:** To test the hypothesis that umbilical cord blood-derived CD34+ hematopoietic stem cells (HPSC), cord tissue-derived CD90+ and CD105+ mesenchymal stem cells (MSC) vary with bronchopulmonary dysplasia (BPD).

**Methods:** We conducted a prospective longitudinal study at a large birth center (Prentice Women's Hospital in Chicago, IL). Premature infants (*N* = 200) were enrolled in 2:1:1 ratio based on gestational age (GA): mildly preterm (31–32 weeks), moderately preterm (29–30 weeks), and extremely preterm (23–28 weeks). Cord blood (CB) and cord tissues (CT) were collected at birth using commercial banking kits, and analyzed for collection blood volume, tissue mass, CD34+, CD90+, CD105+ counts, and concentrations. Multiplex immunoassay was used to measure 12 cytokines and growth factors in CB plasma of 74 patients. BPD severity was defined according to NIH consensus definitions. Univariate and multivariate regression models were used to identify perinatal covariates and assess associations between stem cell concentrations, cytokines, and BPD outcomes.

**Results:** Of 200 patients enrolled (mean GA = 30 ± 2 weeks), 30 developed mild, 24 moderate, and 19 severe BPD. Concentrations of HPSC and MSC, as measured by %CD34+, %CD90+, and %CD105+ of total cells, increased with degree of prematurity. Collection parameters varied with GA, birth weight (BW), gender, prolonged rupture of membranes, mode of delivery, chorioamnionitis, and multiple gestation. Moderate-severe BPD or death was increased with lower GA, BW, Apgar scores, and documented delayed cord clamping. %CD34+ and %CD90+ were increased with BPD and directly correlated with BPD severity. Severe BPD was positively associated with %CD34+ (beta-coefficient = 0.9; 95% CI = 0.4–1.5; *P* < 0.01) and %CD90+ (beta-coefficient = 0.4; 95% CI = 0.2–0.6; *P* < 0.001) after adjustment for covariates. CB plasma granulocyte-colony stimulating factor (G-CSF) was inversely associated with %CD90+, and decreased with BPD. Below median G-CSF combined with elevated %CD90+ predicted BPD (positive predictive value = 100%).

**Conclusions:** CB and CT collections yielded high concentrations of HPSCs and MSCs in BPD infants, accompanied by low circulating G-CSF. These variations suggest possible mechanisms by which stem cell differentiation and function predict BPD.

## Introduction

Premature infants are at risk for a wide array of health and neurodevelopmental problems. Many complications arise because preterm birth disrupts fetal growth and development, with increased vulnerability of virtually every organ system. Promising therapeutic targets in the era of regenerative medicine are the pluripotent hematopoietic stem cells (HPSC) and mesenchymal stem cells (MSC) derived from cord blood (CB) and cord tissues (CT), respectively. Preterm complications that might be preventable or treatable by stem and progenitor cell therapies include diseases involving inflammatory and vascular pathology, with the most common and serious lung complication among infants born <32 weeks being bronchopulmonary dysplasia (BPD) (1, 2).

Ongoing and current understanding is that reduced bioavailability of circulating stem cells in the neonatal period is responsible for insufficient tissue regeneration leading to BPD (3, 4). More recently, there has been focused interest on the role of paracrine factors secreted by MSCs (5, 6), which populate the Wharton's jelly of umbilical CT. The characteristics of CT-derived MSCs in a large cohort of preterm births has not been previously reported, to our knowledge. Furthermore, the cellular composition and recovery of both HPSCs and MSCs according to neonatal outcome, in particular BPD, is poorly understood. CB banking provides a source of autologous stem cells. However, the composition of bankable cells for potential future use is not well-characterized (7, 8). Another important knowledge gap is whether maternal or perinatal factors modify availability of stem cells. This variation is particularly important in preterm births where exposure to intrauterine inflammation and oxidative stress are heightened due to perinatal complications such as chorioamnionitis, preeclampsia, and placental insufficiency (9–12).

The objective of this study was to determine how compositions of HPSCs and MSCs vary with BPD. We tested the hypothesis that certain antenatal and perinatal covariates, and biochemical factors in cord blood, contribute to the stem cell bioavailability observed with BPD.

## Methods

### Study Design and Patient Enrollment

From February 2014 to January 2016, we conducted a prospective longitudinal study of 200 preterm infants and their mothers at Prentice Women's Hospital in Chicago. Eligible pregnant women admitted to the labor and delivery floor for possible preterm delivery at <32 completed weeks' gestation (32 6/7 weeks or less) were identified. Although all such potential deliveries were screened, enrollment was adjusted at regular 6-month intervals to ensure that the final study sample was balanced in a ratio of approximately 2:1:1 based on 3 gestational age (GA) subgroups: 31–32 weeks, 29–30 weeks, and extremely preterm (EPT; 23–28 weeks). The designation of these subgroups was determined before enrollment began and was based upon the known distribution of preterm births at our institution (Prentice), and that would ensure a balanced representation of GA ranging from 23 to 32 weeks that could be accomplished over a 2-year period. Women were excluded if there were known or suspected fetal congenital anomalies, if they delivered after 32 weeks, or if CB or CT could not be collected. After processing, remaining tissues were commercially banked by Viacord at no cost to the family for up to 18 years per a contractual agreement signed by the parents. Informed consent was obtained from all mothers prior to participation. The study was approved by the Institutional Review Boards at Northwestern University and Lurie Children's Hospital.

### Cord Blood and Tissue Collection

CB was collected using commercially available kits provided by ViaCord (Waltham, MA). Immediately after birth, the delivering physician inserted a sterile needle into the clamped umbilical vein swabbed with ChloraPrep (El Paso, TX). Blood was drained via gravity into a lowered collection bag (Pall Corporation, Port Washington, NY). CT was retrieved from the placenta, swabbed twice with 70% isopropyl alcohol and placed in sterile cups. CB/CT were immediately shipped to ViaCord Processing Lab via 24 h courier service, and analyzed for collection and stem cell recovery parameters (CB volume, CT mass, % viability, CD34+, CD90+, and CD105+ cell counts and concentrations, pre- and post-total nucleated cell counts), and then processed for long-term banking.

### Clinical Data Collection

Data were collected prospectively throughout hospitalization on mothers and babies, using previously published standardized data collection procedures (13–15). BPD was defined according to NIH consensus criteria and included mild, moderate, and severe disease (16). Specifically, mild BPD was defined as oxygen requirement at 28 days and breathing room air at 36 weeks corrected GA; moderate BPD was defined as need for <30% oxygen at 36 weeks corrected GA; severe BPD was defined as the need for >30% oxygen or positive pressure ventilation (invasive or non-invasive positive pressure mechanical ventilation, or continuous positive airway pressure) at 36 weeks corrected GA. Seven infants died in the neonatal period, all accompanied by respiratory failure and were therefore included with the primary outcome as BPD or death.

### Multiplex Immunoassays

At delivery, 74 of the 200 patients had a separate sample of cord blood collected (at random) into EDTA tubes at the time of CB/CT collection. The blood was centrifuged (3,000 rpm for 10 min) and plasma was separated and stored at −80°C until assay. Multiplex immunoassay was performed on a panel of 12 angiogenic growth factors and cytokines (Human Angiogenesis/Growth Factor Panel, HAGP1MAG-12K, EMD Millipore, MA). The methods employed Luminex magnetic bead format and have been described previously (14).

### Statistical Analysis

Continuous variables were reported as mean and standard deviation (SD) or median and interquartile range (IQR), and compared using student's *t*-test or analysis of variance (ANOVA) for normally distributed variables and non-parametric tests (Wilcoxon Rank-Sum or Kruskall-Wallis) as appropriate. Categorical variables were compared using Chi-square or Fisher's exact tests. To assess linear associations between stem cell concentrations and BPD outcomes, multivariate linear regression models were used to determine beta-coefficients of log-transformed values, using no BPD as the reference group. Models were adjusted for covariates of BPD and CB/CT collection identified by univariate analysis using a significance threshold of *P* < 0.05. Multiplex biomarker data were analyzed by linear regression models of log-transformed values on %CD34+, %CD90+, and %CD105+. The sample size was determined using historical collection data from term births (*N* > 23,000; average GA = 40.4 weeks) and more recent pilot data from preterm births (*N* = 60; average GA = 32.3 weeks) both provided by Viacord. The mean difference in %CD34+ cells between the two groups was 0.43. Estimating a similar difference with BPD in the preterm group, 200 patients would provide at least 0.90 power with alpha = 0.05, taking into account multiple comparisons and adjustment for 10–20 covariates in the multivariate model.

## Results

Maternal and infant characteristics of the cohort according to BPD or death are shown in Table 1. GA, birthweight (BW), and Apgar scores were different between the two groups, while maternal age, race, and infant gender were similar. About 30% of the births had documented delayed cord clamping, which was higher among infants who developed BPD or died. As expected, median length of stay and other complications of prematurity were higher in the BPD/death group.

**Table 1:** Baseline demographics and clinical characteristics of the cohort.

**Variable**	**No BPD**	**BPD or death**	***P***
	***N* = 120**	***N* = 80**	
Gestational age, mean weeks	31.5 ± 1.4	28.0 ± 2.3	<0.001
Birth weight, grams	1628 ± 314	1088 ± 336	<0.001
**Degree of preterm birth**, ***N*** **(%)**			
31–32 completed weeks	81 (68)	8 (10)	<0.001
29–30 weeks	34 (28)	22 (28)	
23–28 weeks	5 (4)	50 (62)	
**Infant gender**			
Female	59 (49)	42 (53)	0.21
Male	61 (51)	38 (47)	
Apgar 1 min, median (IQR)	7 (4, 8)	5 (3, 6)	<0.001
Apgar 5 min	8.5 (8, 9)	7 (6, 8)	<0.001
**Maternal and Delivery Conditions**			
Maternal age, mean years	32.6 ± 6.2	31.8 ± 5.9	0.39
**Maternal Race**, ***N*** **(%)**			
Black	16 (13)	19 (24)	0.29
White	55(46)	33 (41)	
Hispanic	9 (8)	6 (8)	
Other	40 (33)	22 (28)	
Spontaneous Preterm Labor, *N* (%)	61 (51)	51 (64)	0.07
Antenatal steroids	93 (78)	64 (80)	0.67
Preterm premature ROM	52 (43)	33 (41)	0.77
Prolonged ROM	48 (40)	24 (30)	0.15
Chorioamnionitis	11 (9)	4 (5)	0.41
Preeclampsia	27 (23)	19 (24)	0.84
HELLP syndrome	4 (3)	4 (5)	0.56
**Mode of delivery**			
Vaginal	60 (50)	30 (38)	0.08
C-section	60 (50)	50 (63)	
Multiple gestation	64 (53)	37 (46)	0.33
*In vitro* fertilization	45 (38)	27 (34)	0.59
Delayed cord clamping (documented)	29 (24)	33 (41)	0.01
**Infant outcomes**			
Length of hospitalization, median days (IQR)	35 (26, 45)	65 (45, 95)	<0.001
**BPD severity**, ***N*** **(%)**			
Mild BPD	–	30 (37)	–
Moderate BPD	–	24 (30)	
Severe BPD	–	19 (24)	
Death	–	7(9)	
Patent ductus arteriosus	7 (6)	23 (29)	<0.001
Intraventricular hemorrhage	35 (29)	23 (29)	0.95
Necrotizing enterocolitis	3 (3)	7 (9)	<0.001
Retinopathy of prematurity (stage 2+)	0 (0)	8 (10)	0.001

*BPD or death was defined as having any BPD (mild, moderate or severe BPD) and includes the 7 deaths reported due to respiratory failure. BPD severity categories were based upon the NIH consensus criteria: mild BPD was defined as oxygen requirement at 28 days and breathing room air at 36 weeks corrected GA; moderate BPD was defined as need for <30% oxygen at 36 weeks corrected GA; severe BPD was defined as the need for >30% oxygen or positive pressure ventilation (invasive or non-invasive positive pressure mechanical ventilation, or continuous positive airway pressure) at 36 weeks corrected GA. P-values determined using student's t-test or Wilcoxon Rank-sum for continuous data, and Chi-square or Fisher's exact tests for categorical variables. ROM, rupture of membranes; HELLP, hemolysis, elevated liver enzymes, low platelets*.

Variations in CB and CT characteristics with maternal and perinatal factors are shown in Tables 2, 3. The frequency of successful CB collection (defined as volume that was sufficient for processing and commercial banking by Viacord Processing Lab) was higher among mildly preterm births. However, CB volume per kg BW was higher among EPT infants and with lower BW subgroups. Similar variations were seen with CT collections (Table 3). Rate of recovery of bankable umbilical CT was higher than that of CB (94 vs. 70%, respectively, *P* = 0.02). One hundred and twenty-seven of the 200 (64%) patients had both CB and CT collected. Maternal and perinatal factors that varied with CB collection included GA, BW, infant gender and mode of delivery (Table 2). GA, BW, prolonged rupture of membranes, chorioamnionitis, and multiple gestation varied with CT collection (Table 3).

**Table 2:** Cord blood and CD34+ parameters according to antenatal and perinatal characteristics.

**Variable**	**Patients enrolled (*N*)**	**Cord blood collected *n* (%)**	**Cord blood volume mL/kg median (IQR)**	**Percent CD34 % of CD45+/kg median (IQR)**	**CD34 cell count # cells × 10^**5**^/kg median (IQR)**	**CD34 >1 × 10^**5**^/kg *n* (%)**
**Gestational age subgroup**						
31–32 completed weeks	89	73 (82)	35 (28, 41)	0.34 (0.22, 0.46)	3.17 (1.32, 6.25)	58 (65)
29–30 weeks	56	35 (63)^***^	37 (30, 49)^*^	0.49 (0.27, 1.10)^**^	3.85 (0.89, 6.99)	26 (46)
23–28 weeks	55	31 (56)^***^	46 (37, 57)^***^	0.88 (0.69, 1.27)^***^	2.55 (1.13, 8.65)	23 (42)
**Birth weight subgroup**						
>1,500 g	86	67 (78)	34 (26, 43)	0.30 (0.20, 0.43)	3.58 (1.78, 7.14)	54 (69)
1,000–1,500 g	79	55 (70)	37 (33, 47)^**^	0.58 (0.37, 0.97)^***^	3.74 (1.03, 8.09)	41 (59)
<1,000 g	35	17 (49)^***^	57 (47, 62)^***^	1.19 (0.68, 1.51)^***^	1.38 (0.99, 2.46)^*^	12 (24)
**Gender**						
Female	101	70 (69)	41 (33, 52)	0.39 (0.26, 0.71)	3.17 (1.09, 8.09)	53 (52)
Male	99	69 (70)	36 (28, 43)^**^	0.45 (0.27, 0.89)	3.17 (1.31, 6.62)	54 (55)
**Preterm labor**						
No	88	63 (72)	37 (31, 47)	0.37 (0.23, 0.60)	3.12 (1.13, 5.42)	49 (56)
Yes	112	76 (68)	38 (30, 46)	0.47 (0.27, 0.89)	3.46 (1.22, 8.72)	58 (52)
**Antenatal steroids**						
No	43	30 (70)	39 (32, 49)	0.52 (0.23, 1.10)	2.66 (1.09, 7.14)	23 (53)
Yes	157	109 (69)	37 (30, 46)	0.42 (0.26, 0.70)	3.19 (1.21, 7.36)	84 (54)
**Prolonged rupture (>18 h)**						
No	128	94 (73)	40 (31, 49)	0.42 (0.23, 0.87)	2.67 (0.98, 5.44)	67 (52)
Yes	72	45 (63)	35 (28, 40)	0.42 (0.29, 0.65)	4.67 (1.88, 10.35)^**^	40 (56)^*^
**Chorioamnionitis**						
No	185	128 (69)	38 (31, 47)	0.45 (0.26, 0.84)	3.12 (1.09, 6.25)	96 (52)
Yes	15	11 (73)	35 (28, 41)	0.39 (0.32, 0.43)	9.23 (2.86, 11.08)^*^	11 (73)
**Preeclampsia**						
No	154	100 (65)	37 (30, 46)	0.43 (0.27, 0.81)	3.68 (1.30, 8.77)	79 (51)
Yes	46	39 (85)	40 (33, 50)	0.37 (0.23, 0.87)	2.55 (0.97, 4.58)	28 (61)
**Mode of delivery**						
Vaginal	90	62 (69)	35 (27, 41)	0.41 (0.23, 0.70)	3.15 (1.30, 7.14)	50 (56)
Cesarean	110	77 (70)	43 (33, 51)^***^	0.50 (0.27, 0.87)	3.17 (1.05, 7.36)	57 (52)
**Twin/triplet**						
No	99	70 (71)	37 (30, 43)	0.37 (0.21, 0.77)	2.94 (0.90, 4.01)	51 (52)
Yes	101	69 (68)	41 (32, 51)	0.46 (0.33, 0.83)	4.01 (1.84, 9.23)	56 (55)
**Delayed cord clamping**						
No	98	98 (100)	38 (32, 49)	0.42 (0.26, 0.84)	3.44 (1.23, 7.78)	78 (80)
Yes	41	41 (100)	37 (30, 43)	0.49 (0.26, 0.77)	2.76 (0.99, 5.51)	29 (71)

* *P < 0.05*;

** *P < 0.01*;

*** *P < 0.001*.

*Continuous variables reported as median (IQR), using Wilcoxon Rank-Sum or Kruskal-Wallis tests to compare groups against the referents of mildly PT, birth weight >1,500, female gender, vaginal delivery, or absence of each covariate (No). Categorical variables compared using Chi-square or Fisher's exact tests*.

**Table 3:** Cord Tissue and MSC parameters according to antenatal and perinatal characteristics.

**Variable**	**Cord tissue collected** ***n* (%)**	**Cord tissue weight** ** grams/kg** ** median (IQR)**	**Percent CD90** ** % of CD45^**−**^/kg** ** median (IQR)**	**Percent CD105** ** % of CD45^**−**^/kg** ** median (IQR)**
**Gestational age subgroup**				
31–32 completed weeks	84 (94)	9 (6, 14)	51.4 (45.5, 59.7)	3.6 (1.9, 8.6)
29–30 weeks	53 (95)	11 (7, 17)^*^	65.4 (58.0, 76.2)^***^	6.8 (3.1, 12.4)^*^
23–28 weeks	51 (93)	15 (9, 21)^***^	96.4 (78.8, 123.4)^***^	6.0 (3.0, 15.0)^**^
**Birth weight subgroup**				
>1,500 g	82 (95)	10 (6, 14)	50.3 (45.3, 55.3)	3.9 (1.8, 9.7)
1,000–1,500 g	72 (91)	10 (7, 18)	72.2 (65.1, 79.7)^***^	4.5 (2.7, 9.6)
<1,000 g	34 (97)	15 (9, 24)^**^	117.3 (99.9, 137.2)^***^	7.1 (3.8, 15.0)^**^
**Gender**				
Female	95 (94)	10 (6, 16)	66.9 (51.7, 89.3)	4.1 (2.2, 9.8)
Male	93 (94)	11 (6, 18)	58.7 (43.3, 75.9)	6.3 (2.5, 11.3)
**Preterm labor**				
No	83 (94)	10 (6, 15)	62.3 (50.7, 80.6)	5.0 (2.6, 9.8)
Yes	105 (94)	12 (7, 17)	63.9 (51.0, 85.6)	4.7 (1.9, 12.4)
**Antenatal steroids**				
No	43 (100)	11 (6, 15)	60.3 (48.0, 90.7)	4.8 (2.5, 13.3)
Yes	145 (92)	10 (6, 17)	63.9 (52.3, 81.0)	4.7 (2.4, 9.8)
**Prolonged rupture (>18 h)**				
No	120 (94)	10 (6, 16)	65.8 (52.4, 90.9)	4.9 (2.5, 12.3)
Yes	68 (94)	12 (7, 18)^*^	58.7 (50.6, 73.7)	4.5 (2.3, 8.8)
**Chorioamnionitis**				
No	173 (94)	10 (6, 17)	64.7 (51.4, 85.6)	4.8 (2.4, 11.0)
Yes	15 (100)	11 (6, 15)^*^	53.8 (49.3, 55.6)	3.2 (2.5, 8.1)
**Preeclampsia**				
No	146 (95)	11 (6, 17)	60.8 (50.3, 84.0)	4.7 (2.4, 11.2)
Yes	42 (91)	10 (6, 19)	67.0 (56.4, 85.0)	4.2 (2.3, 10.8)
**Mode of delivery**				
Vaginal	86 (96)	12 (7, 18)	58.2 (50.3, 75.2)	4.7 (1.8, 9.9)
Cesarean	102 (93)	10 (6, 15)	66.2 (53.6, 89.3)^*^	4.8 (2.6, 11.3)
**Twin/triplet**				
No	97 (98)	14 (10, 21)	60.3 (50.1, 81.0)	4.0 (1.8, 8.1)
Yes	91 (90)^*^	8 (5, 12)^***^	64.7 (53.6, 84.1)	7.1 (3.0, 13.4)^**^

* *P < 0.05*;

** *P < 0.01*;

*** *P < 0.001*.

*Continuous variables reported as median (IQR), using Wilcoxon Rank-Sum or Kruskal-Wallis tests to compare groups against the referents of mildly PT, birth weight >1,500, female gender, vaginal delivery, or absence of each covariate (No). Categorical variables compared using Chi-square or Fisher's exact tests*.

Per kilogram BW, the median percent of hematopoietic (CD45+) cells that were CD34+ (HPSCs) was higher with decreasing GA and BW (Table 2). Absolute number of CD34+ cells/kg was decreased with extremely low birthweight (ELBW), prolonged rupture of membranes, and chorioamnionitis. MSC parameters were measured according to percent CD90+ and CD105+ of the CD45- cell fraction in CT (Table 3). %CD90 was highest in the EPT and ELBW groups and with C-section deliveries. %CD105 was increased with lower GA, lower BW, and multiple gestation.

Table 4 shows percentage of CD34+, CD90+, and CD105+ cells according to BPD and death. %CD34+/kg was higher among infants who later developed any BPD (mild, moderate or severe disease or death) and further increased among infants who had moderate-severe BPD. Similarly, %CD90+ cells/kg was higher with BPD, and appeared to increase with BPD severity. Except for a slight increase with severe BPD, there were no significant associations with %CD105+. In multivariate linear regression analysis of log-transformed cell percentages, BPD was associated with increased %CD34+ and %CD90+ after adjustment for all significantly associated covariates of BPD and CB and CT collections identified in Tables 1–3. After adjustment for all levels of BPD severity and death in the linear models, increased %CD90+ was most strongly associated with severe BPD (beta-coefficient = 0.41; *P* < 0.001).

**Table 4:** Variations in percentage of CD34+, CD90+, and CD105+ cell lines in umbilical cord blood and cord tissue with BPD.

	**Cord blood-derived HPSCs**			**Cord tissue-derived MSCs**				
**Outcome at** ** 36 weeks CGA**	***N***	**%CD34 cells/kg** ** of CD45+** ** Median (IQR)**	**%CD34** ** Beta-coefficient (95% CI)**	**N**	**%CD90 cells/kg** ** of CD45-** ** Median (IQR)**	**%CD90** ** Beta-coefficient (95% CI)**	**%CD105 cells/kg** ** of CD45-** ** Median (IQR)**	**%CD105** ** Beta-coefficient** ** (95% CI)**
**BPD (any)**								
None	94	0.4 (0.2, 0.5)	REF	112	54.4 (467.0, 64.0)	REF	4.3 (1.9, 9.7)	REF
Yes	45	0.9 (0.6, 1.2)^***^	0.7 (0.3, 0.9)^***^	76	90.6 (72.2, 115.0)^***^	0.2 (0.1, 0.3)^***^	5.1 (3.1, 12.9)	0.1 (−0.4, 0.6)
**BPD/death**								
None	94	0.4 (0.2, 0.5)	REF	112	54.4 (47.0, 64.0)	REF	4.3 (1.9, 9.7)	REF
Mild	18	0.7 (0.5, 0.9)^***^	0.5 (0.02, 0.9)^*^	28	82.2 (68.6, 97.3)^***^	0.2 (0.1, 0.3)^**^	4.6 (2.0, 10.6)	0.1 (−0.6, 0.7)
Moderate	13	0.9 (0.6, 1.0)^***^	0.7 (0.2, 1.1)^**^	22	84.4 (67.9, 93.0)^***^	0.1 (-0.01, 0.3)	4.3 (3.5, 13.1)	0.1 (−0.5, 0.8)
Severe	10	1.1 (0.7, 1.9)^***^	0.9 (0.4, 1.5)^**^	19	122.9 (92.5, 152.6)^***^^a^	0.4 (0.2, 0.6)^***^	6.5 (3.9, 15.0)*^†^	−0.1 (−0.9, 0.7)
Death	4	1.0 (0.6, 1.5)^*^	0.7 (−0.01, 1.4)	7	118.2 (53.7, 137.2)^***^	0.3 (0.1, 0.5)^**^	5.1 (3.2, 15.1)	0.3 (−0.7, 1.3)

*BPD (any) was defined as having any BPD (mild, moderate or severe BPD) and includes the 7 deaths reported due to respiratory failure. Subsequently, multivariate analysis of all 5 distinct outcomes (BPD/Death) with “None” group as the reference, are detailed in the last 5 rows: Mild BPD was defined as oxygen requirement at 28 days and breathing room air at 36 weeks corrected GA; moderate BPD was defined as need for <30% oxygen at 36 weeks corrected GA; severe BPD was defined as the need for >30% oxygen or positive pressure ventilation (invasive or non-invasive positive pressure mechanical ventilation, or continuous positive airway pressure) at 36 weeks corrected GA*.

* *P < 0.05*,

** *P < 0.01*.

*** *P < 0.001 vs. control (None)*.

a *P < 0.05 vs. mild and/or moderate BPD*.

*Percentage of cells per kg birth weight are reported as median levels and compared using Kruskal-Wallis and post-hoc Dunn tests for multiple comparisons. Beta-coefficients were determined by multivariate linear regression models of the BPD outcomes on log-transformed % cells per kg birth weight, using “none” as the reference group and further adjusted for gestational age, Apgar scores, delayed cord clamping, infant gender, mode of delivery, prolonged rupture of membranes (>18 h), chorioamnionitis, and multiple gestation*.

Despite higher %CD34+ in BPD infants, the total CD34+ counts recovered were not significantly different from non-BPD infants (3.1 ×10^5^ cells/kg vs. 3.4 ×10^5^ cells/kg, respectively; *P* = 0.97). However, over 75% of infants in both groups had CD34+ cell recovery thresholds above 1 ×10^5^ cells/kg (72/94 non-BPD and 35/45 BPD patients).

### Comparison With Viacord Standards

Collection data for 23,196 CB and 12,060 CT collections over a 1-year period (2013–2014) was provided by Viacord, in which the mean GA was 40.4 weeks. The average volume of blood collected was twice that of the Prentice preterm sample (98.7 vs. 50.0 mL), and CT collection was 50% higher (26.6 vs. 17.9 g). However, stem cell counts were comparable and slightly higher for %CD34+ (Viacord average = 0.42 vs. Prentice = 0.78%), CD90+ (72.5 vs. 89.6%), and CD105+ (7.0 vs. 9.8%). CB and CT viability were comparable to Viacord standards (81.0 and 48.7%, respectively).

### Cytokines, Chemokines, and Growth Factors in Cord Blood Plasma

For 74 of the births, archived CB plasma was available for multiplex immunoassay. Table 5 shows the levels of each analyte, according to BPD status. Angiopoietin-2 was directly correlated with BPD, while granulocyte-colony stimulating factor (G-CSF) was negatively correlated with BPD risk. In multiple linear regression of the 12 analytes on each stem cell line (Tables 6–8), G-CSF was negatively correlated with %CD90+ (Beta = −0.09; *P* = 0.03). Biomarker on %CD90+ associations were modified after stratification by BPD such that in the absence of BPD, %CD90 was significantly associated with G-CSF (*P* = 0.01), Angiopoietin-2 *P* = 0.04), IL-8 (*P* = 0.01), HGF (*P* = 0.03), and FGF-2 (*P* = 0.03). We further explored the interaction effects of low G-CSF (defined as <71 pg/mL, based upon the below-median level for this cohort which is also consistent with published data on cord blood of infants <32 weeks) (17) combined with elevated %CD90+ (top quartile of the sample distribution) on prediction of BPD. When combined, these 2 conditions resulted in a positive predictive value of 100% (sensitivity = 54.3%, specificity = 100%, AUC = 0.75) in which all 6 cases of low G-CSF with high %CD90+ predicted BPD.

**Table 5:** Multiplex immunoassay results of 12 cytokines, chemokines, and growth factors in cord blood of 73 infants, according to BPD.

**Cord blood analyte:**	**Median [IQR] pg/mL**			**Odds Ratio (95%CI)**
	**All** ** (*N* = 74)**	**No BPD** ** (*N* = 54)**	**Any BPD** ** (*N* = 20)**	
Epidermal Growth Factor (EGF)	10.0 [2.5, 37.9]	5.8 [2.5, 28.3]	21.7 [4.7, 93.5]	0.6 (0.1, 4.5)
Angiopoietin-2 (ANG-2)	4,311 [2,540, 9,613]	3,578 [2512, 6673]	10,147 [4,311, 17,245]^**^	11724.1 (2.6, 5.3 ×10^7^)^*^
Granulocyte-colony stimulating factor (G-CSF)	71.2 [26.9, 273.8]	78.7 [31.0, 238.6]	47.3 [21.5, 5183.9]	0.1 (0.01, 0.6)^*^
Bone Morphogenetic Protein-9 (BMP-9)	256.6 [173.2, 452.0]	247.9 [174.6, 452.0]	271.6 [120.1, 445.1]	0.2 (0.0, 14.0)
Endoglin (ENG)	1143.2 [740.8, 2074.5]	1088.6 [740.8, 2074.3]	1469.9 [750.4, 2206.5]	0.00 (6.2 ×10^−10^, 1.5)
Endothelin-1 (ET-1)	7.0 [2.7, 13.3]	5.5 [2.7, 13.3]	8.8 [5.2, 15.6]	2.8 (0.2, 32.4)
Interleukin-8 (IL-8)	10.7 [4.11, 28.4]	10.5 [3.3, 20.5]	24.0 [5.0, 135.5]^*^	6.4 (0.5, 87.2)
Hepatic Growth Factor (HGF)	357.4 [141.4, 715.7]	294.6 [141.3, 649.4]	445.9 [180.3, 2344.8]	1.1 (0.01, 124.5)
Heparin Binding EGF-like Growth Factor (HBEGF)	32.9 [13.3, 84.4]	28.6 [12.6, 82.5]	57.4 [15.9, 130.1]	0.5 (0.01, 35.9)
Placental Growth Factor (PGF)	2.9 [1.2, 6.7]	2.8 [1.2, 5.7]	5.3 [1.2, 30.5]	2.6 (0.2, 26.6)
Fibroblast Growth Factor-2 (FGF-2)	48.9 [24.8, 105.1]	43.8 [24.2, 80.3]	84.7 [36.0, 167.9]	2.9 (0.2, 41.6)
Vascular Endothelial Growth Factor (VEGF-A)	12.5 [12.5, 60.3]	12.5 [12.5, 60.3]	12.5 [12.5, 48.3]	0.3 (0.04, 1.8)

* *P < 0.05*;

** *P < 0.01*.

*Odds ratios calculated using multivariate logistic regression models of log-transformed biomarkers on any BPD (mild, moderate, severe BPD, or death), adjusted for simultaneous measurement of 12 analytes, gestational age, low Apgar, and birthweight-for-gestational age <10th percentile*.

**Table 6:** Associations of cord blood analytes and cord blood-derived CD34+ hematopoietic stem cells, stratified by BPD outcome.

	**Cord blood analytes on CD34+** **cell concentration beta-coefficient (95% CI)**		
**Analyte**	**All** ** (*N* = 74)**	**No BPD** ** (*N* = 54)**	**Any BPD** ** (*N* = 20)**
EGF	−0.18 (−0.41, 0.06)	−0.20 (−0.53, 0.13)	−0.31 (−1.33, 0.71)
ANG-2	0.03 (−0.55, 0.61)	0.31 (−0.75, 1.37)	−1.68 (−4.54, 1.19)
G-CSF	−0.17 (−0.37, 0.04)	−0.12 (−0.44, 0.20)	−0.04 (−1.25, 1.18)
BMP-9	−0.13 (−0.52, 0.25)	0.04 (−0.81, 0.90)	−0.96 (−3.78, 1.86)
ENG	0.14 (−0.62, 0.90)	−0.09 (−1.15, 0.97)	2.23 (−2.59, 7.06)
ET-1	0.28 (−0.02, 0.57)	0.23 (−0.18, 0.64)	0.41 (−1.25, 2.07)
IL-8	0.12 (−0.22, 0.45)	0.02 (−0.47, 0.52)	0.31 (−1.76, 2.38)
HGF	0.01 (−0.48, 0.50)	−0.29 (−1.01, 0.42)	0.83 (−1.55, 3.21)
HBEGF	0.34 (−0.19, 0.87)	0.71 (−0.18, 1.60)	0.70 (−1.92, 3.31)
PGF	−0.25 (−0.52, 0.02)	−0.48 (−0.98, 0.01)	−0.53 (−2.44, 1.39)
FGF-2	0.00 (−0.26, 0.26)	0.01 (−0.31, 0.33)	−0.64 (−2.45, 1.17)
VEGF-A	−0.13 (−0.30, 0.03)	−0.18 (−0.40, 0.05)	−1.17 (−15.08, 12.73)

*Beta-coefficients calculated by multivariate linear regression models of the 12 analytes (log-transformed) on %CD34^+^ per kg (log-transformed), adjusted for gestational age, low Apgar, and birthweight-for-GA <10th percentile. Models were stratified by any BPD (yes/no)*.

## Discussion

In this prospective study of 200 preterm births, we found that compositions of CD34+ HPSCs and CD90+ MSCs were increased in premature infants who developed BPD as compared with those who did not. HPSC and MSC percentages appeared to increase with degree of prematurity, a finding that has previously been reported with HPSCs (18–21). After adjustment for GA and other covariates of BPD, preterm birth, and CB/CT collection, the direct associations between BPD, CD34+, and CD90+ tissue concentrations per BW were significant even when taking into account severity of BPD and death in the multivariate linear regression models. Our findings may have important implications for the potential utility and ongoing research efforts in stem and progenitor cell therapies for the prevention and management of BPD.

Recent initiatives have emphasized the importance of developing novel therapies to prevent BPD, and to reduce the morbidity and mortality associated with established disease. A recent Canadian workshop group of neonatologists acknowledged cell-based therapies as promising for BPD (22), and highlighted that successful translation requires careful consideration of antenatal, perinatal, and postnatal factors. They and others also emphasize the need for evidence-based approaches in designing and executing these studies (23, 24). Our study provides epidemiologic evidence that HPSCs and MSCs present at birth vary in concentration among preterm infants who later develop BPD. While some of the variability could be attributed to differences in GA, we took this into account *a priori* by balancing enrollment of GA subgroups, such that a larger proportion of non-extremely preterm infants (non-EPT; GA range 29–32 weeks) was included. As such, one-fourth of all moderate-severe BPD cases in this cohort were non-EPT infants. After adjustment for these and other antenatal and perinatal covariates, the associations with %CD34+ and %CD90+ remained positive. In fact, after stratification of the adjusted models by EPT and non-EPT infants, the associations with BPD remained significant within each strata for both %CD34 (*P* = 0.005 and *P* = 0.006 for EPT and non-EPT groups, respectively) and %CD90 (*P* = 0.003 and *P* = 0.010).

Direct associations with BPD were seen with CD90+ MSCs and severe BPD (Table 4), in which the linear regression findings (beta-coefficient = 0.41) can be interpreted as a roughly 1.5-fold increase in %CD90+ with severe BPD as compared with no BPD. Although we can only speculate about mechanism, there are several reasons why MSCs might be upregulated in infants at risk for BPD. Firstly, MSCs may be recruited or maintained differentially near umbilical circulation, in response to intrauterine stressors such as inflammation (25) or hypoxia-ischemia (26). Secondly, deviations in stem cell differentiation and fate in response to intrauterine stress or epigenetic modifications (27, 28) might have implications for the postnatal capabilities of lung regeneration if MSC are not mobilized from Wharton's jelly at birth. Thirdly, overabundance of MSCs may contribute to ongoing lung injury (29). These MSCs may lack ability to secrete certain proangiogenic and anti-inflammatory cytokines necessary for tissue repair. Recent studies of the role of paracrine functions of MSCs in attenuating BPD support this last hypothesis (5, 6). Our preliminary multiplex data (Table 7) suggest a potential interaction effect of abnormally high %CD90+ with low G-CSF (a glycoprotein typically produced by MSCs and responsible for stem cell mobilization) (30) that further support inherent MSC dysfunction with BPD. If overabundance of MSCs in CT reflects MSC dysfunction, then %CD90+ and plasma G-CSF levels may serve as important predictors. Further delineation of these profiles could guide management as strategies of MSC replenishment and/or their associated microenvironment are developed (31).

**Table 7:** Associations of cord blood analytes and cord tissue-derived CD90+ mesenchymal stem cells, stratified by BPD outcome.

	**Cord blood analytes on CD90+** **cell concentration beta-coefficient (95% CI)**		
**Analyte**	**All** ** (*N* = 74)**	**No BPD** ** (*N* = 54)**	**Any BPD** ** (*N* = 20)**
EGF	0.02 (−0.07, 0.11)	0.05 (−0.03, 0.14)	0.00 (−0.46, 0.46)
ANG-2	−0.08 (−0.30, 0.15)	−0.28 (−0.55, −0.01)^*^	0.22 (−1.07, 1.51)
G-CSF	−0.09 (−0.17, −0.01)^*^	−0.11 (−0.19, −0.03)^*^	−0.25 (−0.80, 0.29)
BMP-9	−0.02 (−0.17, 0.13)	0.08 (−0.14, 0.30)	0.38 (−0.88, 1.64)
ENG	0.07 (−0.22, 0.36)	0.27 (−0.00, 0.54)	−0.85 (−3.02, 1.31)
ET-1	0.00 (−0.11, 0.12)	0.06 (−0.04, 0.17)	−0.25 (−0.99, 0.50)
IL-8	0.12 (−0.01, 0.24)	0.18 (0.06, 0.31)^**^	0.07 (−0.86, 1.00)
HGF	0.16 (−0.03, 0.35)	0.21 (0.03, 0.39)^*^	0.33 (−0.74, 1.40)
HBEGF	−0.06 (−0.27, 0.14)	−0.22 (−0.45, 0.01)	−0.10 (−1.27, 1.08)
PGF	−0.03 (−0.13, 0.08)	−0.03 (−0.16, 0.10)	0.25 (−0.61, 1.10)
FGF-2	−0.10 (−0.20, −0.00)	−0.09 (−0.71, −0.01)^*^	−0.09 (−0.91, 0.72)
VEGF-A	−0.02 (−0.09, 0.04)	−0.01 (−0.07, 0.05)	0.13 (−0.42, 0.67)

* *P < 0.05*;

** *P < 0.01*.

*Beta-coefficients calculated by multivariate linear regression models of the 12 analytes (log-transformed) on %CD90+ per kg (log-transformed), adjusted for gestational age, low Apgar, and birthweight-for-GA <10th percentile. Models were stratified by any BPD (yes/no)*.

In recent years, there has been relatively less focus on the role of HPSCs as potential therapeutic targets for BPD. Our findings suggest that potential utility of cord blood-derived HPSCs and other progenitor cells should continue to be an area of ongoing focus, particularly for BPD prevention. For example, increased availability of HPSCs at birth may be a sign of delayed or inhibited stem cell differentiation, or increased mobilization of dysfunctional stem cells as a compensatory response to intrauterine stress (3). As such, the shift toward undifferentiated cells at birth may serve as a marker that treatment with associated paracrine factors may aid in attenuation of early lung injury (32).

Although our study was not aimed at determining feasibility of CB/CT collections in EPT infants, our results demonstrated a higher than anticipated successful recovery rate when collection was attempted. We considered several perinatal factors that might influence collection in this preterm cohort. Stem cell recovery appeared higher with inflammatory complications such as chorioamnionitis and prolonged rupture of membranes. This finding supports underlying mechanisms of the innate immune system, in which CD34+ stem cell populations are mobilized in the setting of intrauterine inflammation (9). In contrast, there was a non-significant trend toward decreased CD34+ with preeclampsia (Table 2), a finding that supports previous studies in preeclamptic pregnancies (33). Knowledge of the peripartum factors that influence stem cell bioavailability is important as we develop potential therapies in patients whose preterm birth is mediated by chorioamnionitis, preeclampsia and other inflammatory and vascular conditions.

MSCs derived from umbilical cord Wharton's jelly are highly undifferentiated with higher multipotency than bone-marrow derived cells. Typically recruited to areas of hypoxic-ischemic tissue injury, MSCs play a role in regenerative healing through their anti-inflammatory and angiogenic properties (34). Thus, increased MSCs at birth may be an indicator of advanced or chronic intrauterine vascular compromise resulting in fetal growth restriction, a complication linked specifically to long-term end-organ vascular dysfunction characteristic of BPD (35). Although increased, it remains unclear whether these MSCs effectively participate in tissue healing (36). In fact, recent evidence suggests that secretory and paracrine functions of MSCs may be more important determinants in the pathophysiology of BPD and other neonatal vascular diseases (37, 38). Hence, increased MSC concentrations may serve as markers for diseases that respond to exosomal and conditioned media therapies. CD90 remains a well-known marker of umbilical CT-derived MSCs. Also known as Thy-1, CD90 is capable of producing and secreting proangiogenic, antiapoptotic, cytokine, and growth-stimulating factors (39). Less is known about the role of CD105 (endoglin), but its expression on MSCs has recently been associated with increased osteogenic and relatively less differentiation potential than CD90 (40). This may explain the differential associations between these two MSC markers with BPD and the cytokine profiles.

While several of our banking parameters were comparable to Viacord standards of the larger term infant sample, the utility of banked CB/CT for autologous transfusion in EPT infants remains to be determined. While the volumes of collection/kg were higher, the overall amounts were only about half of the term infant average. With respect to HPSCs, 35 of 45 (78%) of infants with BPD who had CB collected had CD34+ cell counts >1 ×10^5^ per kg, which is considered favorable for transplantation but this is measured according to BW and not weight at time of treatment. Ongoing research to characterize and optimize clonogenic potential of these cells is needed (20). Engraftment and functional potential of HPSCs and MSCs derived from preterm infants is poorly understood and requires further investigation. In addition, consideration of a larger profile of cytokines is warranted, as our preliminary findings on the pattern of cytokines (Tables 5–8) suggest that BPD may be associated with impairment in the transition from the innate immune to the adaptive immune response, mediated by T-lymphocytes, to therapeutic strategies directed against cytokines or their receptors. Specifically angiopoietin-2, IL-8, and G-CSF, along with others reported in Tables 6–8 according to their cell-specific patterns with BPD, have been shown to vary in human models of neutropenic sepsis and death and may serve as predictors of response to therapy (41, 42).

**Table 8:** Associations of cord blood analytes and cord tissue-derived CD105+ mesenchymal stem cells, stratified by BPD outcome.

	**Cord blood analytes on CD105+** **cell concentration beta-coefficient (95% CI)**		
**Analyte**	**All** ** (*N* = 74)**	**No BPD** ** (*N* = 54)**	**Any BPD** ** (*N* = 20)**
EGF	0.14 (−0.27, 0.55)	0.26 (−0.32, 0.84)	−0.15 (−1.54, 1.25)
ANG-2	0.57 (−0.45, 1.59)	0.42 (−1.47, 2.31)	1.93 (−2.00, 5.86)
G-CSF	−0.06 (−0.42, 0.29)	−0.12 (−0.69, 0.45)	−0.20 (−1.86, 1.47)
BMP-9	0.02 (−0.66, 0.71)	−0.47 (−2.00, 1.05)	0.57 (−3.30, 4.43)
ENG	−0.97 (−2.31, 0.36)	−0.57 (−2.45, 1.31)	−3.38 (−9.99, 3.24)
ET-1	0.15 (−0.36, 0.67)	0.09 (−0.64, 0.83)	0.28 (−1.99, 2.56)
IL-8	−0.08 (−0.67, 0.50)	0.08 (−0.80, 0.97)	−0.37 (−3.21, 2.48)
HGF	0.17 (−0.69, 1.02)	0.48 (−0.80, 1.75)	−0.48 (−3.75, 2.79
HBEGF	−0.15 (−1.08, 0.78)	−0.90 (−2.48, 0.68)	0.57 (−3.02, 4.16)
PGF	−0.01 (−0.48, 0.46)	0.61 (−0.27, 1.49)	−0.12 (−2.75, 2.51)
FGF-2	−0.17 (−0.62, 0.29)	−0.22 (−0.79, 0.34)	0.70 (−1.79, 3.19)
VEGF-A	−0.03 (−0.32, 0.25)	−0.18 (−0.58, 0.22)	0.08 (−1.58, 1.74)

*Beta-coefficients calculated by multivariate linear regression models of the 12 analytes (log-transformed) on %CD105+ per kg (log-transformed), adjusted for gestational age, low Apgar, and birthweight-for-GA <10th percentile. Models were stratified by any BPD (yes/no)*.

Other limitations of this study include that we could not evaluate cause-and-effect mechanisms by which increased stem cells are associated with BPD. It is important to consider stem cell banking as an opportunity to further our research efforts in this field, by conducting functional and expansion assays, paracrine studies of gene and protein expression, and developing animal models to track the fate and function of banked stem cells. Although we considered multiple antenatal and perinatal factors, larger multicenter studies are needed to take into account other factors that contribute to patient heterogeneity, such as racial and genetic differences, maternal environmental influences, and differences in postnatal course which undoubtedly interact with early predictors in the pathophysiology of multifactorial BPD.

In conclusion, HPSCs and MSCs are increased at birth in infants who develop BPD. Our findings support early mechanisms by which stem cells might play a role in the pathogenesis of BPD. HPSCs and MSCs may serve as markers by which to guide upcoming trials of stem cell therapies in premature infants. Although much research is still needed to determine the utility of cord-derived HPSCs and MSCs, successfully collected tissues provide an important avenue for ongoing investigation.

## Data Availability Statement

The datasets generated for this study are available on request to the corresponding author.

## Ethics Statement

The studies involving human participants were reviewed and approved by Northwestern University Institutional Review Board and Lurie Children's Hospital Institutional Review Board. Written informed consent to participate in this study was provided by the participants' legal guardian/next of kin.

## Author Contributions

KM and SC were responsible for creating the overall study design and objectives, approval and oversight of protocols for patient enrollment, data collection, analysis and interpretation of results, wrote the first draft of the manuscript, and revised subsequent drafts. JS provided her expertise in designing the protocols for patient recruitment, tracking, data collection, and biospecimen handling and processing. RB designed the panel and methods for multiplex immunoassays and provided his expertise in analysis and interpretation of the stem cell data. KF-G and MK assisted with the initial study design and protocols for tissue collection, processing, and banking. LE designed the standardized protocols for umbilical cord tissue collection, analysis, and processing. WG contributed his expertise in maternal-fetal medicine and epidemiology to design the methods for patient recruitment, clinical, and biospecimen data acquisition in labor, and delivery, interpretation and analysis of results.

### Conflict of Interest

KM and SC received an investigator-initiated research grant from Viacord. KF-G is Director of Medical and Scientific Affairs at ViaCord a Perkin Elmer Company. MK is Chief Scientific Officer at ViaCord a Perkin Elmer Company. The remaining authors declare that the research was conducted in the absence of any commercial or financial relationships that could be construed as a potential conflict of interest.
